# Multiplexed PCR-Free Detection of MicroRNAs in Single Cancer Cells Using a DNA-Barcoded Microtrough Array Chip

**DOI:** 10.3390/mi10040215

**Published:** 2019-03-27

**Authors:** Nayi Wang, Yao Lu, Zhuo Chen, Rong Fan

**Affiliations:** 1 Department of Biomedical Engineering, Yale University, New Haven, CT 06520, USA; wangnayi@gmail.com (N.W.); luyao@dicp.ac.cn (Y.L.); zhuo.chen@yale.edu (Z.C.); 2 Yale Cancer Center, Yale University School of Medicine, New Haven, CT 06520, USA; 3 Yale Stem Cell Center, Yale University School of Medicine, New Haven, CT 06520, USA; 4 Human and Translational Immunology Program, Yale University School of Medicine, New Haven, CT 06520, USA

**Keywords:** single-cell analysis, microRNA, DNA barcoding, microtrough arrays

## Abstract

MicroRNAs are a class of small RNA molecules that regulate the expression of mRNAs in a wide range of biological processes and are implicated in human health and disease such as cancers. How to measure microRNA profiles in single cells with high throughput is essential to the development of cell-based assays for interrogating microRNA-mediated intratumor heterogeneity and the design of new lab tests for diagnosis and monitoring of cancers. Here, we report on an in situ hybridization barcoding workflow implemented in a sub-nanoliter microtrough array chip for high-throughput and multiplexed microRNA detection at the single cell level. The microtroughs are used to encapsulate single cells that are fixed, permeabilized, and pre-incubated with microRNA detection probes, each of which consists of a capture strand complementary to specific microRNA and a unique reporter strand that can be photocleaved in the microtroughs and subsequently detected by an array of DNA barcodes patterned on the bottom of the microtroughs. In this way, the measurement of reporter strands released from single cells is a surrogate for detecting single-cell microRNA profiles. This approach permits direct measurement of microRNAs without PCR amplification owing to the small volume (<1 nL) of microtroughs. It offers high throughput and high multiplexing capability for evaluating microRNA heterogeneity in single cells, representing a new approach toward microRNA-based diagnosis and monitoring of complex human diseases.

## 1. Introduction

MicroRNAs (miRNAs), an important class of small RNAs, are involved in nearly all biological processes including cell proliferation, differentiation, and metabolism [1,2,3,4,5]. They regulate messenger RNA (mRNA) expression level or function via mRNA translation repression or RNA degradation [1]. Dysregulated apoptosis or abnormal proliferation of cells, often implicated in human cancers, can be induced by miRNAs [6,7,8]. Deletion of tumor-suppressive miRNAs (TS-miRs) or over expression of oncogenic miRNAs (onco-miRs) have been identified in several types of cancer [9,10,11]. However, due to the complex regulatory mechanism of miRNAs in modulating target mRNAs, how to understand the exact role of each miRNA in every cell and how to translate such findings to a miRNA biomarker assay for clinical diagnostics is a challenging task. In particular, due to non-genetic cell-cell heterogeneity in development or tumors [3,5,12], it is required to conduct multiplexed miRNA measurements at the level of single cells. To date, despite the recent progress in single-cell small RNA sequencing [13,14], how to measure a panel of miRNAs in a highly-multiplexed manner from hundreds to thousands of single cells such that intratumor miRNA heterogeneity can be quantitatively examined with high statistical power, is still difficult. Moreover, how to perform such assays in the clinical settings via a rapid on-site test is a challenge that needs to be overcome to bring single cell miRNA profiles to cancer diagnosis and classification.

A majority of existing microRNA detection technologies such as RT-PCR or microarray-based miRNA assays are not suitable for single cell analysis [15,16]. The PreAmp workflow (ABI/Thermo Fisher, Waltham, MA, USA) adds an intermediate step between RT and real-time PCR to pre-amplify cDNA prior to qPCR detection [17]. However, it is mainly for single-plex real-time PCR, and each PCR tube contains one cell, which is a low throughput and labor intense process. AmpliGrid microwell array-based single cell capture chip allows for medium throughput miRNA assay on 48 single cells per chip [18]. However, this technology is costly and has very limited multiplexing capability. To execute the conventional RNA processing workflow at the level of single cells requires miniaturized fluid handling systems to conduct multi-step RNA extraction, chemical modification, purification, and amplification, for example, in a Fluidigm C1™ (South San Francisco, CA, USA) Single-Cell Auto Prep System [19]. It is one of the most powerful systems for complex biochemical workflow at the nanoliter scale and together with the BioMark HD System allows for processing of ~96 single cells for a panel of genes [20]. However, this device is costly, and the workflow is time consuming. A microfluidic chip was reported for analyzing >1000 single cells for on-chip qRT-PCR detection of 1 or 2 miRNA biomarkers simultaneously but is still yet to demonstrate highly multiplexed detection. As of today, these technologies either lack the ability of a highly multiplexed assay or only offers low to medium throughput. Moreover, most of these methods are time-consuming, costly, or clinically impractical.

Here, we report on a sub-nanoliter microtrough array chip for multiplexed and high-throughput miRNA detection at the single-cell level. The microtroughs are used to encapsulate single cancer cells that were fixed, permeabilized, and pre-incubated with a set of uniquely designed miRNA detection probes, each of which consists of a DNA oligomer capture strand complementary to the miRNA of interest and the other half is a unique reporter strand that can be cleaved inside the microtrough upon UV exposure. The cleaved reporter strands are then captured by an array of DNA barcodes pattern on the bottom of each microtrough such that the detection of reporter strands with known sequences is a surrogate for detecting miRNAs in single cells via in situ hybridization and barcoding. This approach had demonstrated the limit of detection for miRNAs down to the single-cell level without PCR amplification due in part to the small volume (<1 nL) of microtroughs. It offers high throughput and high multiplexing capability for examining miRNA heterogeneity in single cancer cells. 

## 2. Materials and Methods

### 2.1. Fabrication of Flow Patterning Device for Creating DNA Barcode Microarrays

DNA barcode array slides were fabricated in-house using a flow patterning technology we reported previously [21,22,23,24,25,26]. The microfluidic chip used to perform flow patterning was fabricated with polydimethylsiloxane (PDMS) using soft lithography techniques [27,28,29]. The master molds for replicating PDMS were fabricated on silicon wafers using deep reactive ion etching (DRIE) [30,31]. After overnight treatment of the master mold with trimethylchlorosilane (TMCS) (Aldrich, St. Louis, MS, USA), PDMS prepolymer and curing agent, mixed in 10:1 ratio, were poured into the silicon master, degassed, and then cured in a convection oven at 80 °C for 3 h. Afterwards, the PDMS layer was peeled off from the mold, cut into rectangular slabs with desired dimensions, and access holes punched for fluidic insets and outlets. Before using them, the PDMS slabs were cleaned in 50% (vol/vol) 2-isopropanol and then in DI water for 30 min. At last, the PDMS chips were bound to a poly-l-lysine microarray slide (Thermo-Scientific) for flow patterning. 

### 2.2. Fabrication of Sub-Nanoliter Microtrough Array Chips

The sub-nanoliter microtrough array chips were also fabricated with PDMA using soft lithography. The master mold used herein was made of SU8 2010 negative photoresist (~15 μm in thickness) lithographically patterned on a silicon wafer. Following the similar procedures described above, the resultant microtrough array PDMS chip contains 14 columns, each of which consists of 550 microtroughs. Thus, each device has 7700 microtroughs in total for single cell isolation. Each microtrough measures 15 μm in width, 10 μm in depth, 1850 μm in length, and 20 μm in spacing.

### 2.3. Flow Patterning of DNA Oligomers for Detecting The Reporter Probes

DNA probes were immobilized onto a high-quality poly-L-lysine (PLL)-coated glass slide to form DNA barcode arrays using a flow patterning technology. Added to the inlet of the microfluidic channel was 2 μL of capture DNA probe prepared at 500 μM, and each of the microchannels was loaded with a different DNA barcode oligomer (see DNA sequences in Supplementary Tables S1 and S2). In order to increase the uniformity and loading density of the DNA probe, 30% dimethyl sulfoxide (DMSO) was used to prepare these DNA solutions [32]. The external pressure used for flow pattering was between 1–3 psi. Once the DNA oligo solutions were flowed through to the reach outlets, the entire device comprising the PLL-coated glass slide and the flow patterning PDMS chip still remained assembled and directly moved into a desiccator overnight to allow for slow evaporation and uniform deposition of DNA oligomers on the surface of the glass slide. After complete evaporation, the PDMS layer was carefully removed by submerging the whole device in the blocking buffer (1% BSA/PBS). Finally, the glass slide was rinsed with deionized water and gently dried with forced nitrogen gas. 

### 2.4. Design of MicroRNA Capture and Reporter Probes

In our multiplex assay panel, 13 endogenous miRNAs were chosen because they are either known to be involved in multiple human cancers or specifically expressed in human myeloid leukemia cells, including the cell line used in this study (e.g., miR-16, miR-21, mir-146a and mir-221) [33,34,35,36,37]. In order to obtain high detection sensitivity with fluorescence, we chose to use a biotinylated probe that can be detected with ultra-bright phycoerythrin-conjugated streptavidin reporter (SA-PE). In this study, we designed a set of 13 DNA oligomers, each of which contains a sequence for detecting miRNA via hybridization and a distinct sequence as reporter that can be photocleaved by exposure to ultraviolet (UV) light and then detected by flow-patterned DNA barcodes. 

### 2.5. Population Level Multiplexed MicroRNA Assay

To validate the performance of these probes at the population level for the same cell line and in the settings equivalent to single-cell microchip assays, we used the conventional pin-spotting technique to print all 13 DNA oligomers onto a PLL-coated glass slide for detecting miRNA reporter strands released from a whole population of cells in an Eppendorf tube. A human mononuclear leukemia cell line THP-1 (ATCC) was used as the cancer cell model. THP-1 cells from the culture media were spun down and re-suspended in 0.2 mL of single-cell suspension with phosphate-buffered saline (PBS) in a 1 mL tube. Then, they were fixed using paraformaldehyde (4% in PBS) and permeabilized using 90% methanol. Afterwards, a cocktail of all 13 UV-cleavable miRNA capture/reporter probes was added and incubated for 2 h at 37 °C to allow for in situ hybridization. Then, the cells were washed thoroughly 3 times with the Tris-EDTA-Triton X-100 (TET) buffer (0.05% Tween-20, pH = 8.0) containing 350 mM NaCl. In between, the cells were incubated in TET buffer for 5 min to allow unbound DNA probes to diffuse out and be rinsed off from the interstitial space of the fixed cells. The tube was then placed in a UV exposure unit for ~20 min to cleave and release the reporter probes and then left in a 37 °C incubator overnight. Finally, the supernatant was collected and added on the pin-spotted DNA oligo array on top of which was placed an eight-well (~5 mm in diameter) PDMS slab to confine the fluid such that multiple samples can be assayed on the same DNA microarray slide. After incubation for 1 hour and rinsing with 1% BSA/PBS, SA-PE was used to read out the fluorescence signals using a Genepix 4200A laser microarray scanner (Molecular Devices, San Jose, CA, USA) [24,38]. 

### 2.6. Single-Cell Multiplexed MicroRNA Assay

THP-1 cells were spun down and washed twice with cold Dulbecco’s phosphate-buffered saline (DPBS). First, the cells were re-suspended, fixed with paraformaldehyde (4% in PBS), and incubated for 10 min at 37 °C. The cells were spun down again and rinsed completely with DPBS to remove the paraformaldehyde solution. Secondly, 90% methanol was added into the sample tube and kept on ice for 30min for permeabilization. After half an hour, methanol solution was removed. Third, a cocktail of photocleavable miRNA capture/reporter probes (1 μL each at a concentration of 100 μM, which translates to 100 nanomole per probe) was added into 1 mL of single-cell suspension and incubated at 37 °C for 2 h. Afterwards, cells were spun down and washed with TET buffer six times and re-suspend in 1 mL of fresh TET buffer (0.05% Tween-20 in TE buffer, pH = 8.0, containing 350 mM NaCl). Fourth, single-cell suspension at a density of 0.5 × 10^6^ cell/mL in TET buffer was prepared and loaded into the microtrough array PDMS chip. In order to measure single-cell miRNA profiles, the cells were isolated into a microtrough array by gravity and then fully sealed with the DNA barcode glass slide. The volume of each microtrough is 0.55 nL. The DNA barcode microarray contains immobilized DNA oligomers specifically for hybridization with the reporter strands. 

The detailed procedure is the following. Single-cell suspension already fixed, permeabilized, and incubated with microRNA capture/reporter probes but not yet subjected to UV exposure, was pipetted to the microtrough array chip and then the immobilized DNA barcode slide was placed on top of the PDMS microtrough array, which was further clamped together with a setup shown in Figure 2a. After cell loading, the whole device was then exposed under a UV lamp for ~20 min followed by incubation at 37 °C overnight. The next day, the glass slide was removed from the microtrough array PDMS chip and washed with TET buffer solution several times. A solution of SA-PE diluted by 1:100 in TET buffer was used to generate fluorescent signals. Finally, the dried glass slide was scanned using a laser microarray scanner (Axon^®^ Genepix Professional 4200A, Molecular Devices, San Jose, CA, USA) with a 488 nm laser excitation and 532 nm filtered PMT. Fluorescence intensities of the DNA barcodes can be quantified using the algorithms reported previously. 

### 2.7. Single-Cell FISH and qPCR Validation Experiments

We performed fluorescence in situ hybridization (FISH) on selected miRNAs (miR-16) and compared them to our single cell data. This was conducted using a protocol described by the Dr. Singh group [39] and Dr. Tsourka [40]. In addition, qRT-PCR was also performed to detect the same panel of microRNAs according to Qiagen’s protocol to validate our microchip-based assay. 

## 3. Results

**Figure 1:**
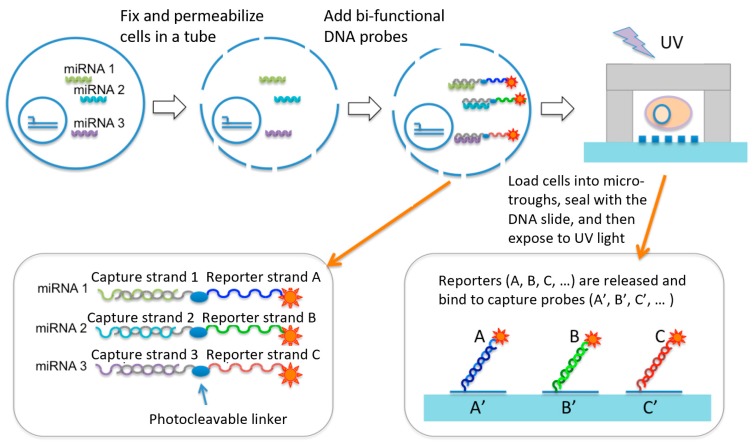
Design of overall workflow. It employs a unique set UV cleavable miRNA capture/reporter probes and combines with a microtrough array chip for single-cell microRNA measurement. This UV cleavable probe contains a photocleavable (PC) covalently linked with two DNA oligomer strands. One is complementary with the miRNA targets and the other is complementary with the DNA barcode probes immobilized on the surface of a poly-L-lysine (PLL)-coaed glass slide.

Our approach (Figure 1) was built upon a single-cell micro-trough miRNA assay chip and the design of a set of UV cleavable miRNA capture/reporter probes that bind to miRNAs of interest via an in situ hybridization process in bulk, but after single-cell isolation in microtroughs are cleaved to release the reporter strands, which are subsequently detected using immobilized DNA barcode microarray in the same microtrough to achieve single-cell level miRNA measurement. Basically, a suspension of live cells in a conventional Eppendorf tube was fixed and permeabilized such that cytosolic miRNAs are fixed inside the cell. MiRNAs of interest were probed by in situ hybridization with a bi-functional DNA probe containing a portion of sequence (called a detection strand) complementary to the miRNA of interest and the other portion being a unique sequence to report the presence of this miRNA (called a reporter strand). In between is a UV cleavable linker. A suspension of cells that have been fixed, permeabilized, and incubated with the miRNA detection/report probes are loaded randomly to a microtrough array chip, sealed with a DNA barcode array slide, and imaged with a motorized light field microscope such that the microtroughs containing single cells can be identified. The DNA barcode array slide has an array of high-density DNA oligomers immobilized and each DNA oligomer is complementary to one of the miRNA reporter strands we designed. After cell isolation into the microtrough array and sealed with the DNA barcode slide, the entire device is placed in a UV exposure unit to cleave the reporter strands, which diffuse across the microtroughs and bind to immobilized DNA barcode spots. Finally, after rinsing the slide to remove unbound reporter probes and non-specific binding, the presentence of specific reporter strands was detected by fluorescence using Streptavidin-PE that binds to biotinylated reporter sequences. Therefore, the approach uniquely converts the detection of intracellular miRNAs into the detection of corresponding reporter strands we designed and can be readily expanded to many other miRNAs of interest. Integrated microfluidics allows for relatively high throughput (~1000 single cells) compared to manual processing of single cells for miRNA assay or sequencing. It also has the ability to multiplex to a level of tens of miRNA co-detected per cell to delineate single-cell miRNA expression heterogeneity. 

**Figure 2:**
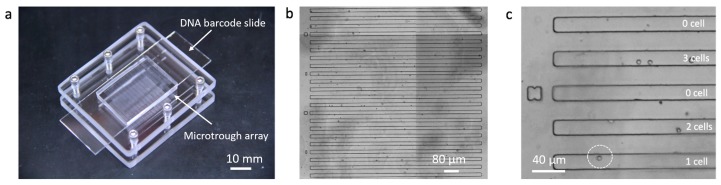
Sub-nanoliter microtrough array chip. (**a**) Fully assembled device; (**b**) microtroughs imaged with a motorized microscope; (**c**) enlarged view of a region of interest showing single or multiple THP-1 cells trapped in individual microtroughs.

A sub-nanoliter microtrough array chip was fabricated with PDMS using soft lithography. Human THP-1 cells were directly pipetted onto the surface of the microtrough array and allowed to be isolated by gravity into the microtroughs. Each device contains 7700 microtroughs and this random cell loading can still yield up to ~2800 single cell data points according to Poisson distribution statistics. A fully assembled device after cell loading is shown in Figure 2a. Microtroughs are shown in Figure 2b, each of which measures 15 μm in width, 10 μm in depth, and 1850 μm in length, with a volume of 0.55 nL. The number of cells in each microtrough was counted using an automated motorized optical microscope (Figure 2c) and the cell distribution matrix was later matched with the quantitated DNA barcode image data to extract single-cell miRNA data. 

**Figure 3:**
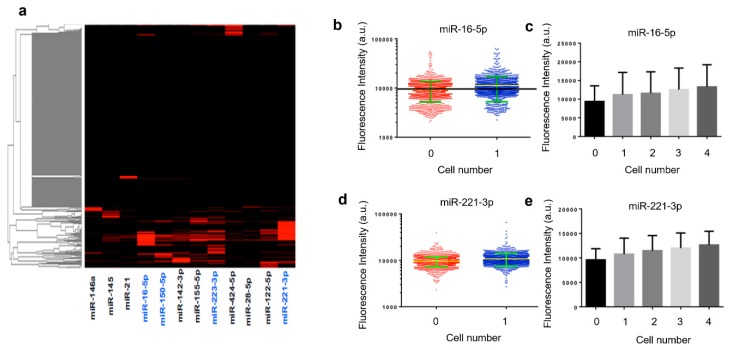
Quantification of single THP-1 cell microRNA detections. (**a**) Heatmap showing the expression of 13 microRNAs detected in single THP-1 cells isolated in microtroughs. The higher intensity indicates the higher expression level of the miRNA of interest. The ones (in blue) shown with relatively high expression and frequency are further validated with population or single cell comparative experiments. (**b**) Single cell expression intensity of miR-16-5p; (**c**) expression level of miR-16-5p increases with cell number in each microtrough; (**d**) single cell expression intensity of miR-221-3p; (**e**) expression level of miR-221-3p increases as cell number in each microtrough increases.

Quantified results from an experiment with human THP-1 cells are shown in Figure 3. The heatmap depicts miRNA profiles across ~1000 single cells identified in a microtrough chip. It was observed that only a fraction of the cells were statistically positive for these microRNAs, although most of these microRNAs are known to be expressed. This may indicate the existence of single-cell miRNA expression heterogeneity but could be due in part to the limit of detection (LOD) our method can provide as of now. Further development could be the inclusion of signal amplification either in situ in the fixed cells or after photocleavage to amplify the reporter strands. MiRNAs (blue) with relatively high expression levels and frequencies in the population of THP-1 cells are further examined in detail and compared with different validation experiments. For example, miR-16-5p and miR-221-3p are shown in scatter plots (Figure 3b,d). Fluorescence intensities quantified directly from raw scanned images show an appreciable increase in single-cell microtroughs as compared to zero cell control microtroughs. It is noted that these plots are in the log scale and the linear scale difference can be readily appreciated for discerning single-cell signals. The outliers in zero cell control data are likely single-cell data points. Due to the low contrast of fixed cells imaged with a low objective power, motorized light field microscope, we expect many missed single-cell microtroughs in the cell detection and counting process. When the intensities from single-cell microtroughs are compared to the microtroughs containing 2, 3, and 4 cells (Figure 3c,e), we observed a steady increase of average fluorescence intensities, indicating the real contribution of single-cell miRNA signals rather than backgrounds. 

**Figure 4:**
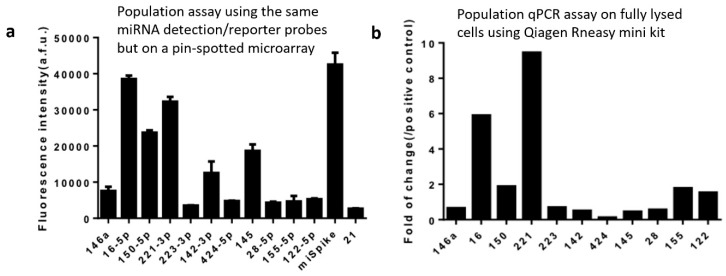
Population validation. (**a**) Population level measurement of microRNAs in THP-1 cells using the same in situ hybridization workflow in Figure 1 and the same reagents including microRNA detection/reporter probes and the DNA barcode array slide except that the UV-cleaved reporter probes were collected in the bulk supernatant and the DNA barcode array was fabricated using conventional pin-spotting. (**b**) qPCR result of THP-1 cell population experiment conducted using the standard cell lysis, RNA extraction, and qPCR workflow rather than in situ hybridization. The y-axis is the fluorescence intensity of certain kinds of miRNAs compared with the positive control U6. The x-axis represents 11 endogenous miRNAs and two controls.

In order to validate our data on the selected miRNAs successfully detected using our technology and workflow, we performed population-level experiments to measure the same panel of miRNAs in THP-1 cells. The first population experiment was performed using the same in situ hybridization workflow and the same reagents including the whole set of 13 miRNA capture/reporter probes and the DNA barcode array slide, except that the UV-cleaved reporter strands were released in the Eppendorf tube and collected in the bulk supernatant and the DNA barcode array was fabricated using conventional pin-spotting. It was found that the most abundant miRNAs detected at the population level were miR-16-5p and miR-221-3p (Figure 4a), which match perfectly single-cell data (Figure 3) where these two are also the top-ranked miRNAs detected at the single-cell level. In order to evaluate the possibility of system bias caused by the specific workflow and reagents we designed in this study, we also performed a population-level validation experiment with the conventional biochemistry workflow using well-established commercialized kits (e.g., Qiagen RNeasy Mini kit) to extract RNAs from the THP-1 cell lysate followed by purification to generate population samples for quantitative RT-PCR detection of the same panel of miRNAs (Figure 4b). The highest-expression miRNAs identified by the conventional methods are again miR-16 and miR-221, which demonstrated the validity of our method via in situ hybridization and barcoding for single-cell miRNA measurement. 

**Figure 5:**
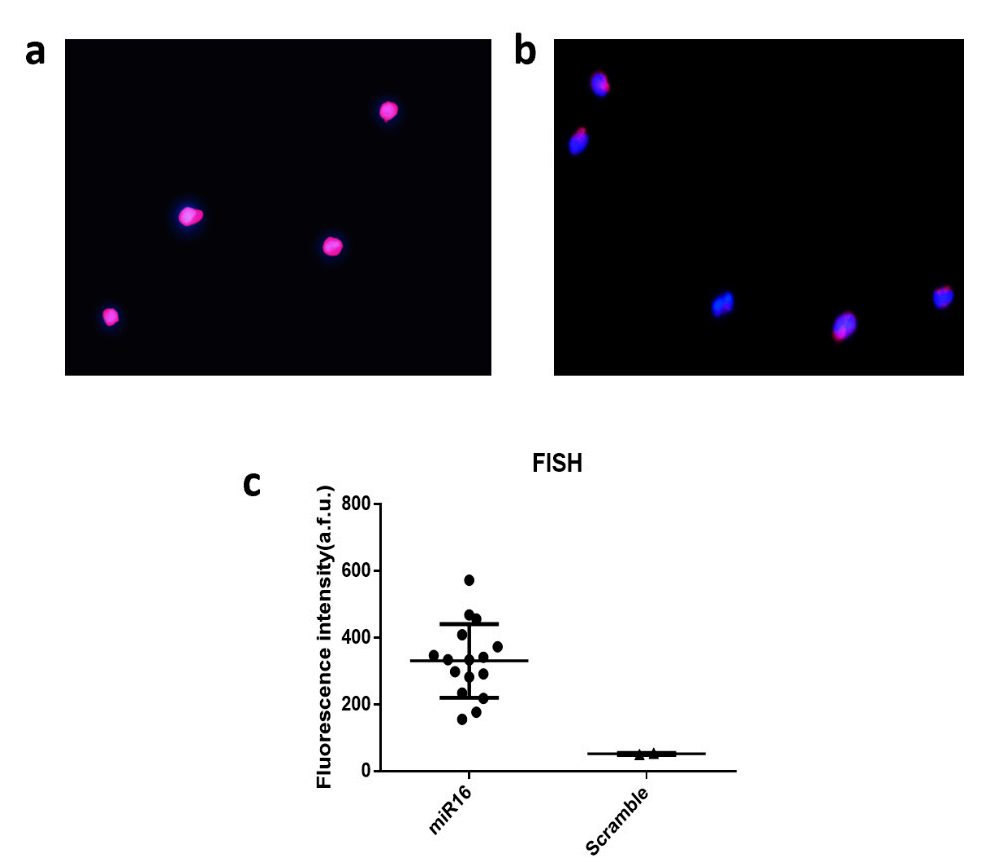
Single-cell fluorescent in situ hybridization (FISH) for single-cell data validation. (**a**) Fluorescence image showing miR-16 detection by fluorescence in situ hybridization (FISH). (**b**) Fluorescence image from negative control using the scrambled sequence; Blue color is for DAPI and pink is for APC to report the FISH signal; (**c**) Quantitative analysis of single-cell miR-16 expression by FISH as compared to scramble. Each dot is a single cell.

To further validate the results at the single-cell level, we conducted single-cell fluorescent in situ hybridization (FISH) measurement on fixed THP-1 cells to detect miR-16 as compared to scramble (negative control). As shown in Figure 5a, the staining for nucleus with 4′,6-diamidino-2-phenylindole (DAPI) and miRNA signals using Allophycocyanin (APC) dye gave rise to bright signals, indicating the expression of miR-16 in these cells. If the FISH probe was modified with a scrambled sequence, the fluorescence intensity for APC is much reduced relative to DAPI (Figure 5b). The expression was further quantified for all single cells in ~10 image frames and the statistics unambiguously confirmed the expression of miR-16 (Figure 5c), which is the most abundant miRNA within our panel we successfully detected in THP-1 cells using our technology and workflow at both single-cell and population levels. 

## 4. Discussions

We demonstrated a novel workflow implemented in a microtrough array device to measure a panel of 13 miRNAs at the single-cell level. It was validated using population DNA microarray assay, population qRT-PCR assay, and single-cell FISH experiment. Although it was demonstrated for selected miRNAs and single-cell level detection limit, the background signal is still high, and the limit of detection is yet to be further increased to improve the performance in the future. To reduce the background of DNA barcode slide, additional blocking reagents such as yeast tRNA and salmon sperm DNA could be used together with 1% BSA/PBS. We noticed the increase of the washing time for cleaning the DNA barcode slide from 4 times to 8 times did not reduce the signal intensity but markedly decreased the background, resulting in increased signal-to-noise ratio. This biochemistry workflow can be improved by designing capture/reporter probes with increased binding efficiency between miRNA targets using hairpin structures. Increasing the incubation time or reaction temperature might be helpful to improve hybridization efficiency and specificity. The dimensions of the microtroughs can be modified to reduce the diffusion distance and increase the binding of probes to miRNAs in a fixed cell. It is known that miRNAs are wrapped by Argonaute (AGO) protein [41,42,43,44], which may decrease the efficiency of in situ hybridization, although miRNA FISH has been well demonstrated, widely used, and commercialized. This issue still exists at least for some miRNAs and may lead to a compounding factor in our data interpretation. For example, miRNAs with low detectable levels in our data could be attributed to low expression but could also be due to inaccessibility of the miRNA sequence complementary to our capture probe. Finally, this work does not require any amplification and has demonstrated single-cell level sensitivity for selected microRNAs in THP-1 cells. We anticipate the use of signal amplification methods such as the amplification of reporter strands either in cell or post photocleavage as well as the amplification of fluorescent signals on the DNA barcode slide could all further improve the performance of this technology. 

In summary, we have developed a new biochemical workflow implemented in a microtrough array chip for single-cell multiplexed microRNA measurement. Recent advances [13,14,45] in the field of single-cell microRNA and mRNA sequencing have demonstrated the potential to co-sequence both small and large RNAs in same single cells and to identify novel regulatory mechanisms^14^ underlying the development of human cancers. Upon the discovery of the key miRNAs involved in such mechanisms, how to reduce the measurement to a targeted miRNA panel but with high throughput is important for translational development of single-cell miRNA assays for clinical applications. Our technology bridges the gap with the ability to multiplex to a dozen and potentially tens of miRNA markers and the throughput required to measure ~1000 or more single cells in parallel, providing a new path toward miRNA-based detection and monitoring of complex human diseases.

## Supplementary Materials

DNA sequences are available online at https://www.mdpi.com/2072-666X/10/4/215/s1, Table S1: Sequence design of 13 microRNA capture/reporter DNA oligomers; Table S2: Sequence of DNA barcodes immobilized on the glass slide.
