# Supplementary material for: Multiplexed PCR-Free Detection of MicroRNAs in Single Cancer Cells Using a DNA-Barcoded Microtrough Array Chip
Source: Micromachines (Basel). 2019 Mar 27;10(4):215. doi: 10.3390/mi10040215 (PMC6523668; doi:10.3390/mi10040215)
Supplement: Supplementary file 1 [file micromachines-10-00215-s001.pdf]

Supplementary Materials

Multiplexed PCR-Free Detection of MicroRNAs in Single Cancer Cells Using a DNA-Barcoded Microtrough Array Chip

Nayi Wang [1], Yao Lu [1], Zhuo Chen [1] and Rong Fan [1,2,3,4,*]

[1] Department of Biomedical Engineering, Yale University, New Haven, CT 06520, USA; wangnayi@gmail.com (N.W.); luyao@dicp.ac.cn (Y.L.); zhuo.chen@yale.edu (Z.C.)
[2] Yale Cancer Center, Yale University School of Medicine, New Haven, CT 06520, USA
[3] Yale Stem Cell Center, Yale University School of Medicine, New Haven, CT 06520, USA.
[4] Human and Translational Immunology Program, Yale University School of Medicine, New Haven, CT 06520, USA
* Correspondence: rong.fan@yale.edu

Received: 8 March 2019; Accepted: 25 March 2019; Published: 27 March 2019

Table S1: Sequence design of 13 microRNA capture/reporter DNA oligomers

1. hsa-miR-146a MI0000477: A′

5' biotin- AAA AAA AAA AAA ATA CGG ACT TAG CTC CAG GAT AAA AAA – PC Linker – AAA AAA GAT ATA TTT TAA ACC CAT GGA ATT CAG TTC TCA /3InvdT/

2. hsa-miR-145 MI0000461: B′

5' biotin AAA AAA AAA AAA ATA GGC ATG ATT CAA TGA GGC AAA AAA – PC Linker – AAA AAA GAT ATA TTT TAA GGG ATT CCT GGG AAA ACT GGA C/3InvdT/

3. hsa-miR-21 MI0000077: C′

5' biotin- AAA AAA AAA AAA GCG ATA GTA GAC GAG TGC AAA AAA – PC Linker – AAA AAA GAT ATA TTT TAT CAA CAT CAG TCT GAT AAG CTA/3InvdT/

4. hsa-miR-16-5p D′

5′ biotin AAA AAA AAA AAA ATA CTC TGA CAT CTC GAC CAT AAA AAA – PC Linker – AAA AAA CGCCAATATTTACGTGCTGCTA /3InvdT/

5. hsa-miR-150-5p E′

5′ biotin AAA AAA AAA AAA ATA GAT ACT GCC ACT TCA CAT AAA AAA – PC Linker – AAA AAA ACTGGTACAAGGGTTGGGAGA /3InvdT/

6. hsa-miR-142-3p F′

5′ biotin AAA AAA AAA AAA ATA CCG TGA ACC TTA CCT GAT AAA AAA – PC Linker – AAA AAA TCCATAAAGTAGGAAACACTACA /3InvdT/

7. hsa-miR-155-5p G′

5′ biotin AAA AAA AAA AAA TGC TCG GGA AGG CTA CTC AAA AAA – PC Linker – AAA AAA CCTATCACGATTAGCATTA /3InvdT/

8. hsa-miR-223-3p H′

5′ biotin AAA AAA AAA AAA ACG CAC CGC AGT TTG GTC AAT AAA AAA – PC Linker – AAA AAA TGGGGTATTTGACAAACTGACA /3InvdT/

9. hsa-miR-424-5p I′

5′ biotin AAA AAA AAA AAA ATC CGA CGC AAC AAT AGG GCA AAA AAA – PC Linker – AAA AAA TTCAAAACATGAATTGCTGCT /3InvdT/

10. hsa-miR-28-5p J′

5′ biotin AAA AAA AAA AAA ACC TGC TCG ACA ACT AGA AGA AAA AAA – PC Linker – AAA AAA TCA ATA GAC TGT GAG CTC CT /3InvdT/

11. hsa-miR-122-5p K′

5′ biotin AAA AAA AAA AAA ACC GCG ACC AGA ATT AGA TTA AAA AAA – PC Linker – AAA AAA AAACACCATTGTCACACTCCA/3InvdT/

12. hsa-miR-221-3p L′

5′ biotin AAA AAA AAA AAA AGC CGA AGC AGA CTT AAT CAC AAA AAA – PC Linker – AAA AAA GAT ATA TTT TAG AAA CCC AGC AGA CAA TGT AGC T /3InvdT/

13. Negative control: Scrameble-miR M′

5′ biotin AAA AAA AAA AAA AAC AGG TTC AGA ATC CTC GAC AAA AAA – PC Linker – AAA AAA GTGTAACACGTCTATACGCCCA/3InvdT

Table S2: Sequence of DNA barcodes immobilized on the glass slide

A 5'- AAA AAA AAA AAA AAT CCT GGA GCT AAG TCC GTA-3'
B 5'-AAA AAA AAA AAA AGC CTC ATT GAA TCA TGC CTA -3'
C 5'- AAA AAA AAA AAA AGC ACT CGT CTA CTA TCG CTA -3'
D 5'-AAA AAA AAA AAA AAT GGT CGA GAT GTC AGA GTA -3'
E 5'-AAA AAA AAA AAA AAT GTG AAG TGG CAG TAT CTA -3'
F 5'-AAA AAA AAA AAA AAT CAG GTA AGG TTC ACG GTA -3'
G 5'-AAA AAA AAA AGA GTA GCC TTC CCG AGC ATT-3'
H 5'-AAA AAA AAA AAT TGA CCA AAC TGC GGT GCG-3'
I 5'-AAA AAA AAA ATG CCC TAT TGT TGC GTC GGA-3'
J 5'-AAA AAA AAA ATC TTC TAG TTG TCG AGC AGG-3'
K 5'-AAA AAA AAA ATA ATC TAA TTC TGG TCG CGG-3'
L 5'-AAA AAA AAA AGT GAT TAA GTC TGC TTC GGC-3'
M 5'-AAA AAA AAA AGT CGA GGA TTC TGA ACC TGT-3'
